# Supplementary material for: The Predictive Value of Gut Microbiota Composition for Sustained Immunogenicity following Two Doses of CoronaVac
Source: Int J Mol Sci. 2024 Feb 23;25(5):2583. doi: 10.3390/ijms25052583 (PMC10931755; doi:10.3390/ijms25052583)
Supplement: Supplementary file 1 [file ijms-25-02583-s001.zip › ijms-2862567-supplementary.pdf]

SUPPLEMENTARY METHODS

Serological test and shotgun metagenomic sequencing

The new version of the iFlash-2019-nCoV NAb kit (chemiluminescent microparticle immunoassay; Shenzhen YHLO Biotech Co, Ltd., Shenzhen, China) was used to test for neutralizing antibody (NAbs) against SARS-CoV-2 receptor-binding domain (RBD). Briefly, serum samples was placed in the sample loading area while a reagent pack containing 2019-nCoV RBD antigen (30KD)-coated paramagnetic microparticles and acrodinium ester-labelled ACE2 conjugate was placed in the reagent loading area. The iFlash system then performed all functions automatically and signals elicited from the chemiluminescent reactions were measured. NAb seroconversion was defined as ≥15 AU/mL.

We extracted total genomic DNA from the stool samples using Qiagen QIAamp DNA stool Mini Kit (Qiagen, Hilden, Germany) in accordance with the manufacturer's instructions. Nextera DNA Library Prep Kit (Illumina, California, USA) was then used to subject the genomic DNA to library preparation for shotgun metagenomic sequencing. In brief, the genomic DNA was first fragmented and tagged with adapter sequences by engineered transposome. Next, limited cycle PCR was used to add the index adapter sequences to these tagged DNA. After amplification, the PCR amplicons were purified using AMPure XP beads (Beckman-Coulter). The quality of the DNA library was then assessed by a Qubit fluorometer (Thermo Fisher Scientific) follwed by a Bioanalyzer (Agilent Technologies). After library preparation, next-generation shotgun metagenomic sequencing was performed using the Illumina NovaSeq 6000 platform (Illumina, San Diego, US) running at paired-end 150 bp, which yielded 10 Gb raw data per sample at the Center for PanorOmic Sciences (CPOS) of the University of Hong Kong (HKU).

SUPPLEMENTARY FIGURES

Figure S1. Comparison of baseline alpha diversity index (Shannon, Simpson, Richness) of gut microbiota between seropositive and seronegative vaccinees of CoronaVac at day 180.

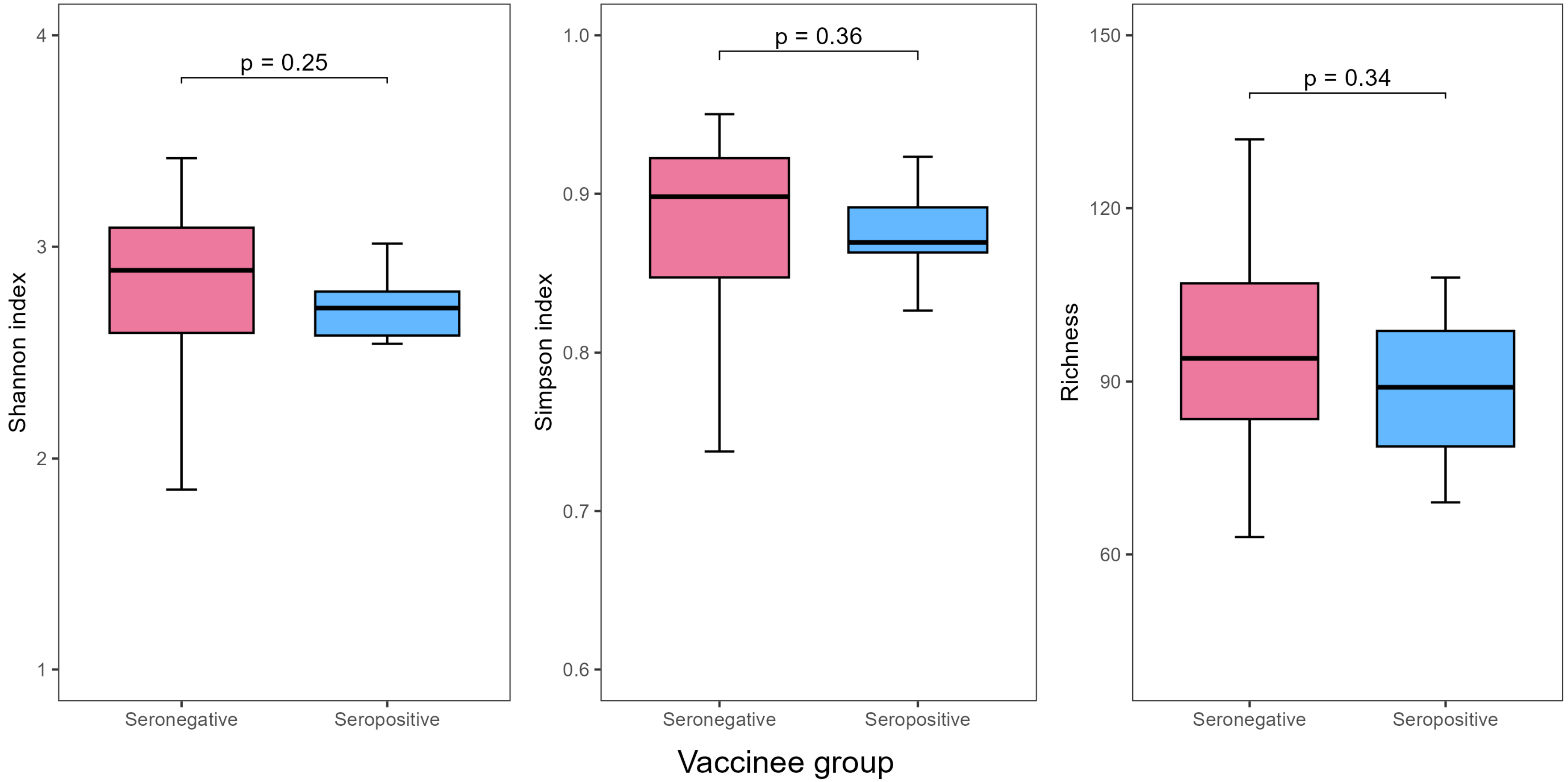

Figure S2. Comparison of baseline beta diversity between seropositive and seronegative vaccinees of CoronaVac at day 180.

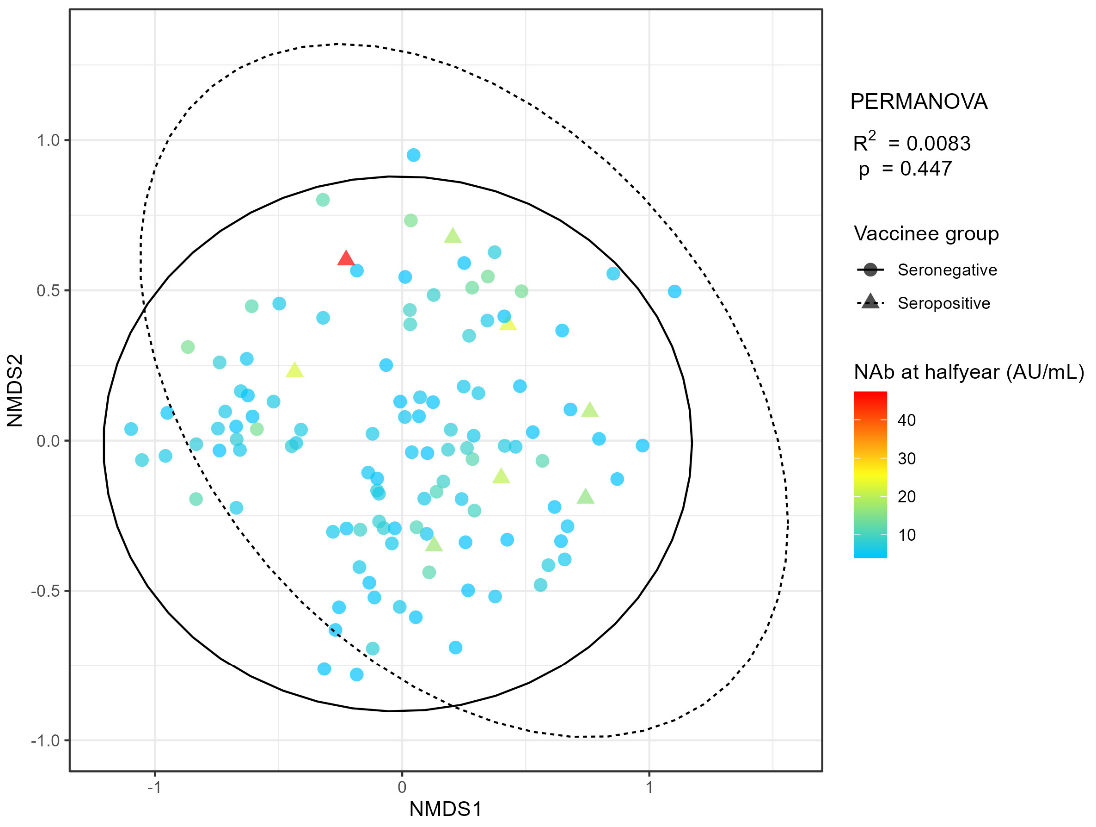

Abbreviations: NAb, neutralising antibody.

Figure S3. Baseline metabolic pathways enriched in seropositive and seronegative vaccinees of CoronaVac at day 180.

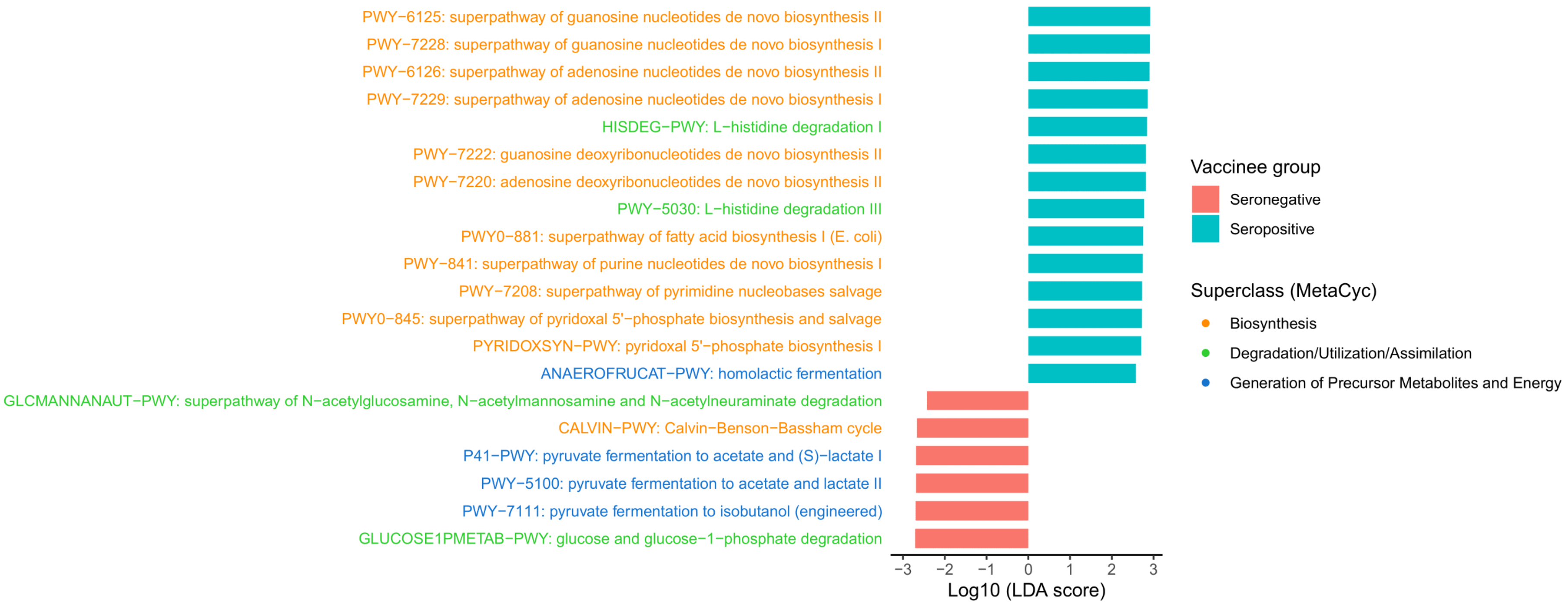

Figure S4. Comparison of baseline alpha and beta diversity index of gut microbiota between CoronaVac recipients with and without any adverse reactions after first dose (**A**,**B**) and second dose (**C**,**D**).

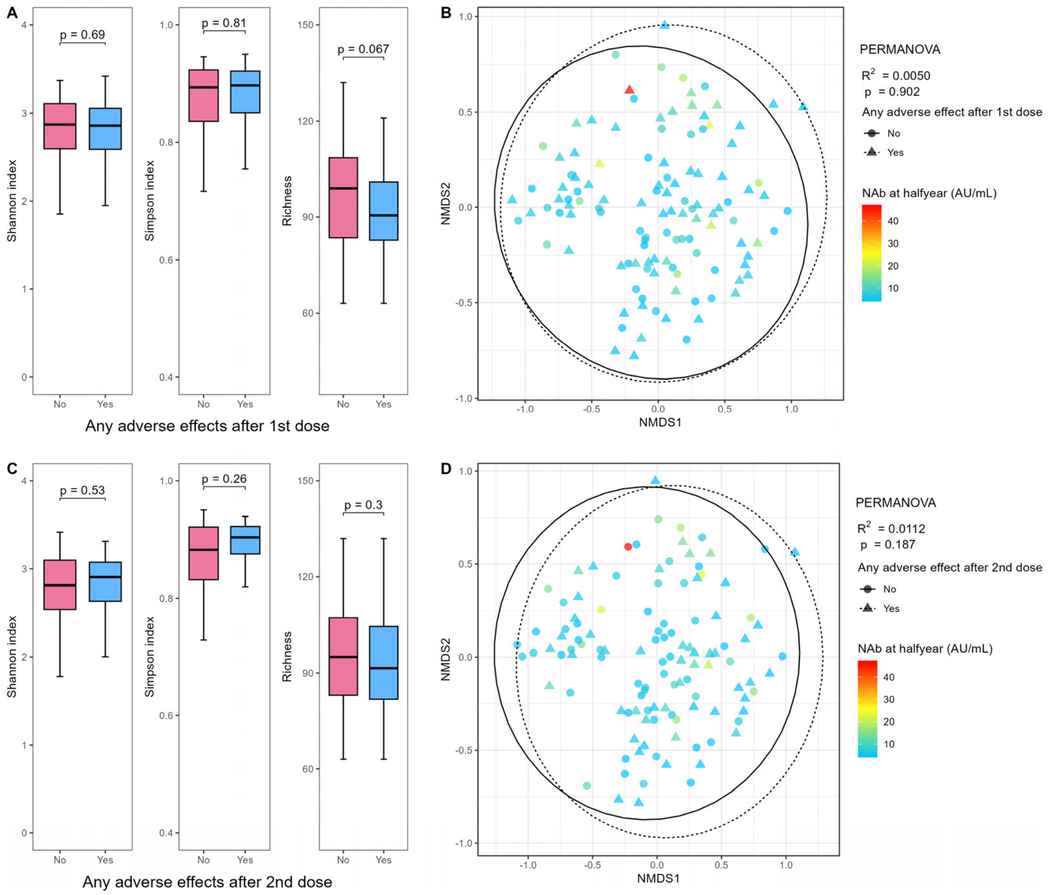

Abbreviations: NAb, neutralising antibody.

Figure S5. Baseline gut bacterial species enriched in CoronaVac recipients with and without any adverse reactions after first dose.

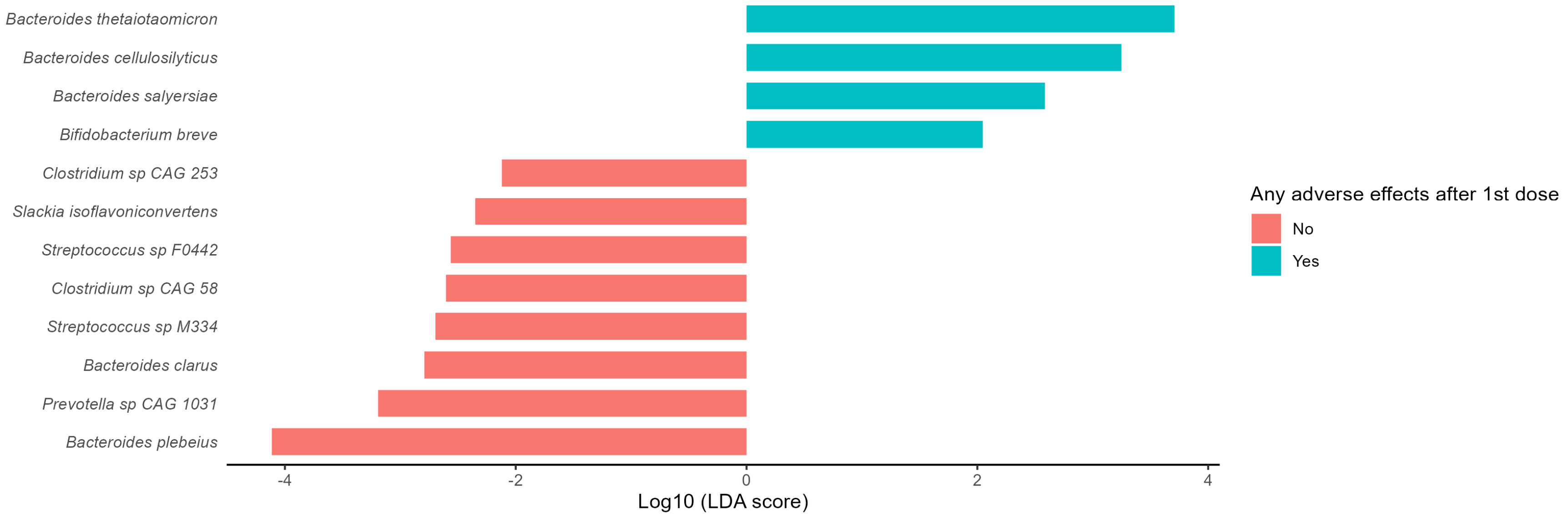

Figure S6. Relative abundances of baseline gut bacterial species among CoronaVac recipients with and without any adverse reactions after first dose**.**

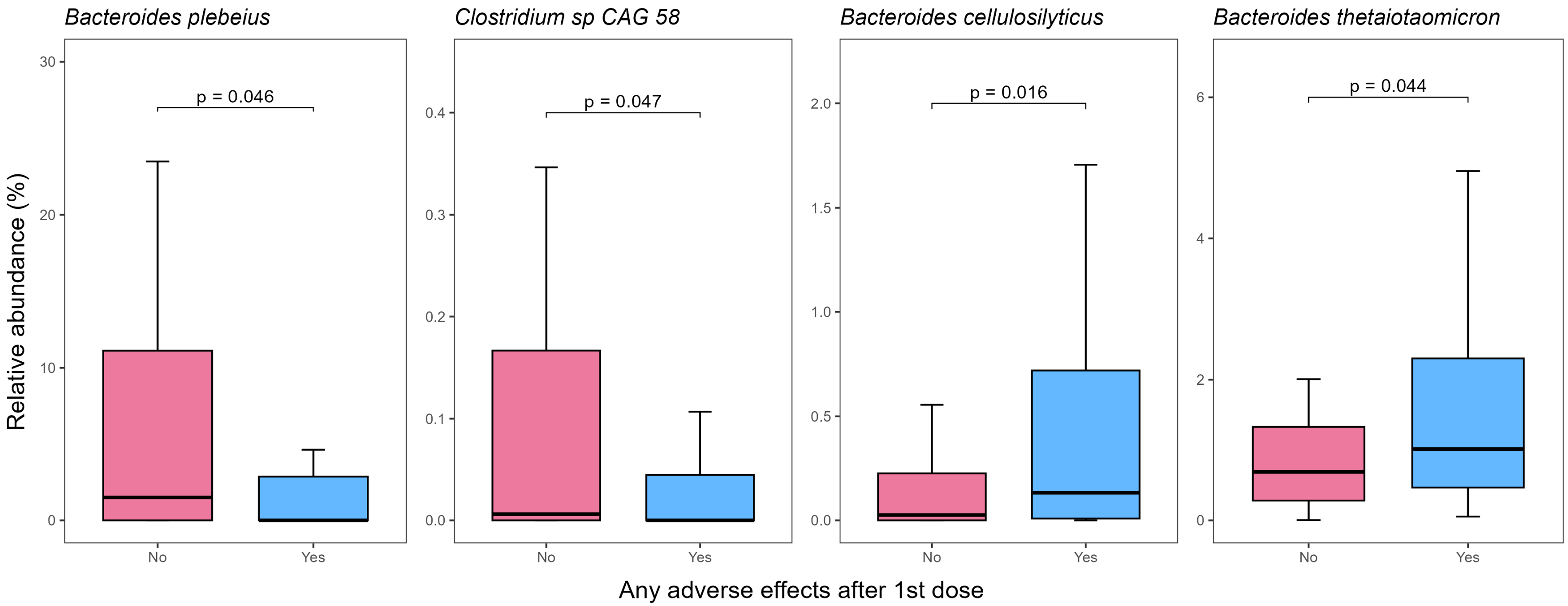

Figure S7. Baseline gut bacterial species enriched in CoronaVac recipients with and without any adverse reactions after second dose.

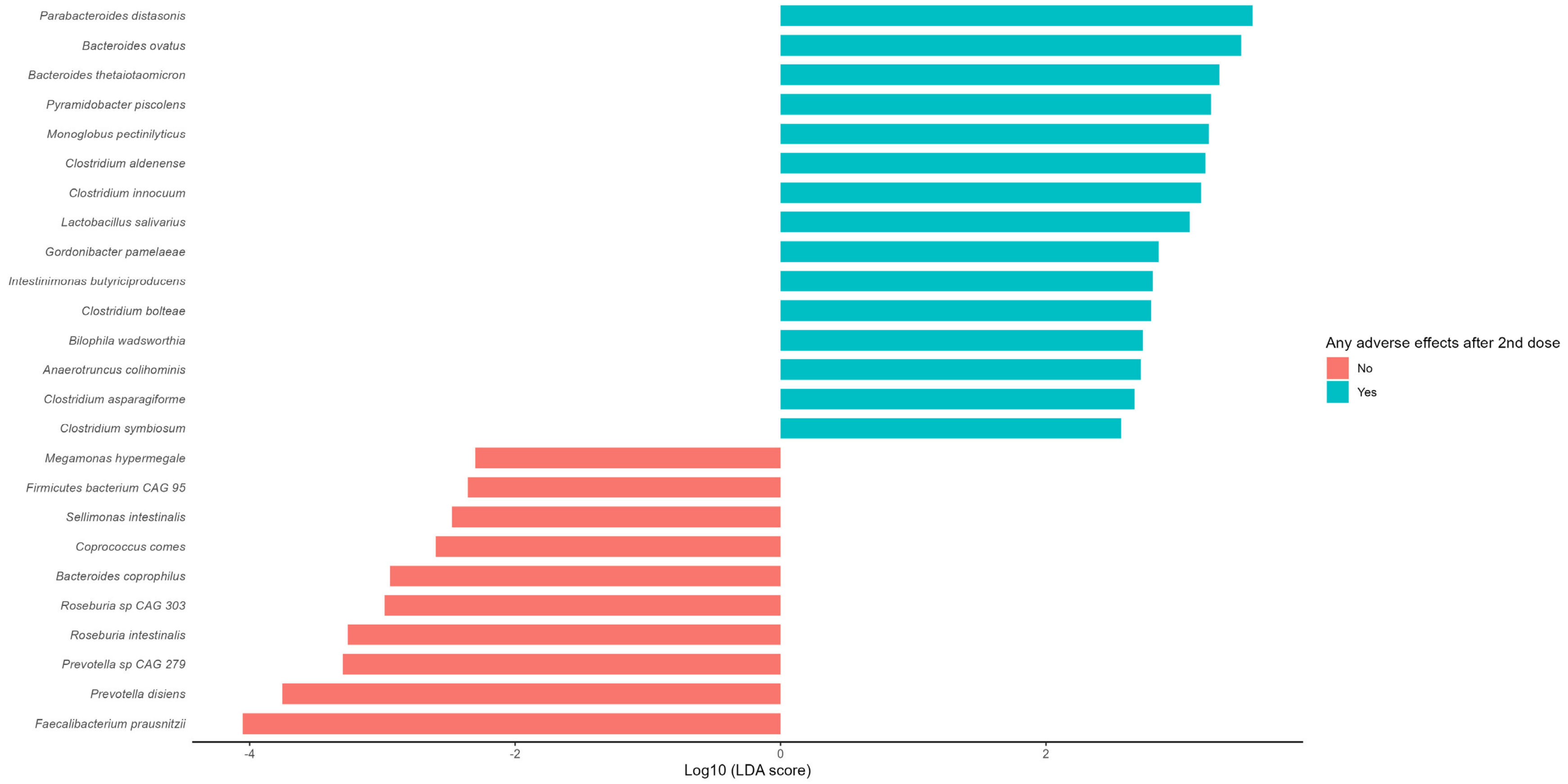

Figure S8. Relative abundances of baseline gut bacterial species among CoronaVac recipients with and without any adverse reactions after second dose**.**

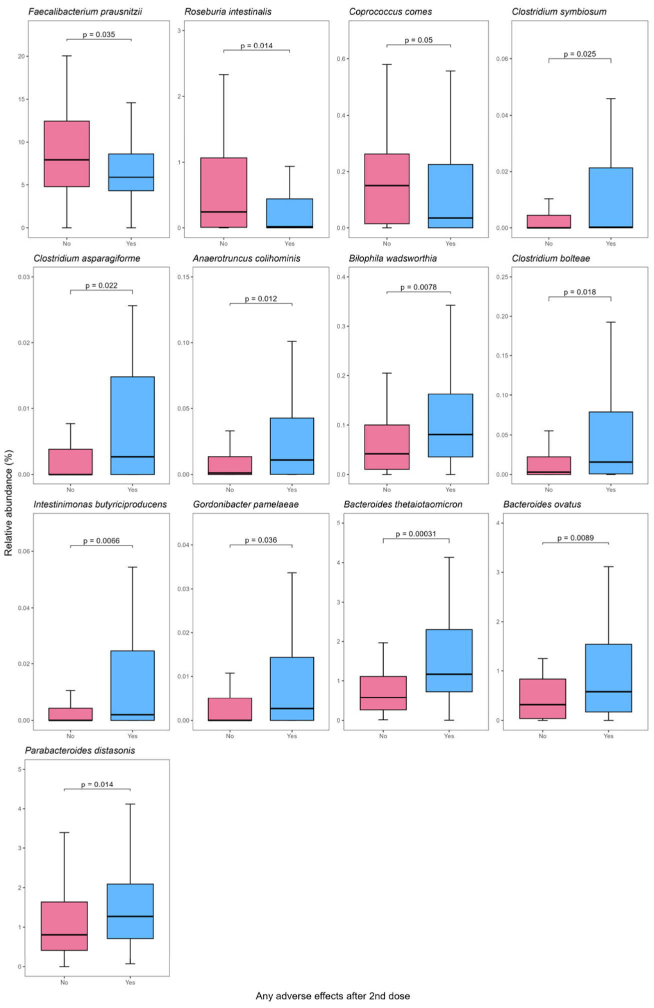

Table S1. Summary of discriminant metabolic pathways and their linear discriminant analysis (LDA) scores between seropositive and seronegative vaccinees.

Abbreviation	Full name	Vaccinee group	LDA score
**Biosynthesis**			
PWY-6125	Superpathway of guanosine nucleotides de novo biosynthesis II	Seropositive	2.918297708
PWY-7228	Superpathway of guanosine nucleotides de novo biosynthesis I	Seropositive	2.909620881
PWY-6126	Superpathway of adenosine nucleotides de novo biosynthesis II	Seropositive	2.902566476
PWY-7229	Superpathway of adenosine nucleotides de novo biosynthesis I	Seropositive	2.858972474
PWY-7220	Adenosine deoxyribonucleotides de novo biosynthesis II	Seropositive	2.815763329
PWY-7222	Guanosine deoxyribonucleotides de novo biosynthesis II	Seropositive	2.815763329
PWY0-881	Superpathway of fatty acid biosynthesis I (E. coli)	Seropositive	2.74922346
PWY-841	Superpathway of purine nucleotides de novo biosynthesis I	Seropositive	2.740703799
PWY-7208	Superpathway of pyrimidine nucleobases salvage	Seropositive	2.724565669
PWY0-845	Superpathway of pyridoxal 5'-phosphate biosynthesis and salvage	Seropositive	2.719245409
PYRIDOXSYN-PWY	Pyridoxal 5'-phosphate biosynthesis I	Seropositive	2.70511082
CALVIN-PWY	Calvin-Benson-Bassham cycle	Seronegative	-2.666094237
**Degradation/Utilization/Assimilation**			
HISDEG-PWY	L-histidine degradation I	Seropositive	2.843740954
PWY-5030	L-histidine degradation III	Seropositive	2.775629447
GLCMANNANAUT-PWY	Superpathway of N-acetylglucosamine, N-acetylmannosamine and N-acetylneuraminate degradation	Seronegative	-2.427773229
GLUCOSE1PMETAB-PWY	Glucose and glucose-1-phosphate degradation	Seronegative	-2.709637614
**Generation of Precursor Metabolites and Energy**			
ANAEROFRUCAT-PWY	Homolactic fermentation	Seropositive	2.5764582
PWY-5100	Pyruvate fermentation to acetate and lactate II	Seronegative	-2.689333461
P41-PWY	Pyruvate fermentation to acetate and (S)-lactate I	Seronegative	-2.689333461
PWY-7111	Pyruvate fermentation to isobutanol (engineered)	Seronegative	-2.698671441

Table S2. Adverse reactions after first and second dose of CoronaVac.

	After 1st dose			After 2nd dose*		
	Seronegative (n = 111)	Seropositive (n = 8)	p-value	Seronegative (n = 108)	Seropositive (n = 8)	p-value
**Total adverse reactions within 7 days after each injection**						
Any	63 (56.8%)	5 (62.5%)	>0.999	51 (47.2%)	1 (12.5%)	0.072
Grade 3 or above[#]	0 (0.0%)	0 (0.0%)	--	0 (0.0%)	0 (0.0%)	--
**Injection site adverse reactions**						
Any	48 (43.2%)	5 (62.5%)	0.464	40 (37.0%)	1 (12.5%)	0.257
Pain	47 (42.3%)	5 (62.5%)	0.295	37 (34.3%)	1 (12.5%)	0.27
Redness	3 (2.7%)	0 (0.0%)	>0.999	8 (7.4%)	0 (0.0%)	>0.999
Swelling	6 (5.4%)	0 (0.0%)	>0.999	4 (3.7%)	0 (0.0%)	>0.999
Itching	3 (2.7%)	0 (0.0%)	>0.999	3 (2.8%)	0 (0.0%)	>0.999
Ecohymoses	0 (0.0%)	0 (0.0%)	--	0 (0.0%)	0 (0.0%)	--
**Systemic adverse reactions**						
Any	45 (40.5%)	1 (12.5%)	0.149	31 (28.7%)	0 (0.0%)	0.107
Fever	3 (2.7%)	0 (0.0%)	>0.999	1 (0.9%)	0 (0.0%)	>0.999
Chills and rigors	2 (1.8%)	0 (0.0%)	>0.999	0 (0.0%)	0 (0.0%)	--
Muscle pain	12 (10.8%)	1 (12.5%)	>0.999	9 (8.3%)	0 (0.0%)	>0.999

Joint pain	2 (1.8%)	0 (0.0%)	>0.999	3 (2.8%)	0 (0.0%)	>0.999
Headache	13 (11.7%)	1 (12.5%)	>0.999	10 (9.3%)	0 (0.0%)	>0.999
Fatigue	31 (27.9%)	1 (12.5%)	0.681	17 (15.7%)	0 (0.0%)	0.602
Nausea	6 (5.4%)	0 (0.0%)	>0.999	2 (1.9%)	0 (0.0%)	>0.999
Vomiting	1 (0.9%)	0 (0.0%)	>0.999	1 (0.9%)	0 (0.0%)	>0.999
Diarrhoea	4 (3.6%)	0 (0.0%)	>0.999	3 (2.8%)	0 (0.0%)	>0.999
Skin rash	4 (3.6%)	0 (0.0%)	>0.999	2 (1.9%)	0 (0.0%)	>0.999
Facial dropping	1 (0.9%)	0 (0.0%)	>0.999	0 (0.0%)	0 (0.0%)	--

* Missing data: 3 after second dose; # Grade 3 or above: severe or life-threatening reactions.
